# Associations between obesity, AD pathology, and neurovascular health in cognitively normal older adults in US POINTER

**DOI:** 10.1002/alz.091590

**Published:** 2025-01-09

**Authors:** Tina E Brinkley, Katelyn R Garcia, Gary F Mitchell, Charles H Tegeler, Aarti Sarwal, John Bennett, Dalane W. Kitzman, Samuel N. Lockhart, Xiaoyan Leng, Laura D Baker, Heather M Snyder, Theresa M. Harrison, Susan M. Landau, Margie J Bailey, Hossam A Shaltout

**Affiliations:** ^1^ Wake Forest University School of Medicine, Winston‐Salem, NC USA; ^2^ Cardiovascular Engineering, (781) 255‐6930, MA USA; ^3^ Wake Forest School of Medicine, Winston‐Salem, NC USA; ^4^ Wake Forest University, Winston‐Salem, NC USA; ^5^ Alzheimer’s Association, Chicago, IL USA; ^6^ University of California, Berkeley, Berkeley, CA USA

## Abstract

**Background:**

Obesity is associated with adverse changes in the structure and function of both the brain and the vasculature and may modify risk for Alzheimer’s disease (AD). However, the degree to which excess total and central adiposity contribute to overall disease burden in late‐life is unclear. We investigated baseline associations between obesity, AD‐related pathology, and neurovascular health in 255 participants enrolled in the U.S. POINTER trial (mean age: 68.2±5.4 years, 53% female, 29% people of color).

**Method:**

Baseline assessments included anthropometry to determine body mass index (BMI) and waist circumference and neuroimaging (MRI and PET) to assess global amyloid burden, entorhinal and temporal metaROI tau burden, hippocampal volume, cortical thickness, and white matter hyperintensity volume (WMHV). Ultrasound, tonometry, and continuous heart rate and blood pressure monitoring were also performed to evaluate the aorta and carotid arteries and assess autonomic function. Linear regression was used to examine cross‐sectional associations between the outcomes of interest adjusting for age, sex, race/ethnicity, education, and site.

**Result:**

The majority of participants were overweight or obese (n = 220, 86.3%, mean BMI: 30.5±5.6 kg/m^2^) or had abdominal obesity (n = 190, 74.5%, mean waist circumference: 104.4±14.4 cm^2^). Overweight and obese individuals were more likely to be Black or Hispanic (p = 0.0028), have less than a college degree (p = 0.0245), and have altered carotid artery properties, including lower characteristic impedance (p = 0.0117), higher pulsatility (p≤0.01), and a larger diameter (p<0.01). Both BMI and waist circumference were positively associated with carotid pulsatility, carotid transmission coefficient, carotid diameter, and aortic normalized impedance, whereas negative associations were found with aortic pulsatility and aorto‐carotid reflection coefficient (all p<0.02). BMI was also positively associated with hippocampal volume (p<0.01). In addition, several neurovascular outcomes were associated with AD‐related pathology, independent of obesity. Notably, higher carotid‐femoral pulse wave velocity, lower baroreflex sensitivity, and lower heart rate variability were associated with greater WMHV (p≤0.01), while higher aortic pulsatility was associated with greater entorhinal and metaROI tau (p = 0.0132).

**Conclusion:**

In older adults at risk for AD, overweight/obesity is associated with altered aortic and carotid structure and function, which likely has important downstream effects on the brain and may impact the development of AD.

